# Microbial Profile and Genetic Polymorphism of Predominant Species in Some Traditional Fermented Seafoods of the Hainan Area in China

**DOI:** 10.3389/fmicb.2019.00564

**Published:** 2019-03-21

**Authors:** Shuaiming Jiang, Chenchen Ma, Qiannan Peng, Dongxue Huo, Wu Li, Jiachao Zhang

**Affiliations:** College of Food Science and Technology, Hainan University, Haikou, China

**Keywords:** high-throughput sequencing, microbial diversity, fermented shrimp, *Lactobacillus*, SNPs

## Abstract

Fermented fish, fermented shrimp and fermented crab are traditionally prepared seafoods that are commonly consumed in the Hainan area in China. We studied the microbial diversity and metabolic pathways in traditional fermented seafoods using high-throughput sequencing technology, and based on our previous research, we also compared the differences between fermented seafood and fermented vegetables. The alpha diversity of fermented seafood was higher than that of fermented vegetables and attained the highest level in fermented shrimp. The dominant genera in fermented seafood were different from those of fermented vegetables. Furthermore, we analyzed the 16S rDNA gene polymorphisms (SNPs) of the same dominant species (*Lactobacillus plantarum* and *Lactobacillus fermentum*) in two fermented environments, which showed that most of the mutations occurred in fermented vegetables and that fermenting environment might be the major factor for these mutations. This research provides us with new insights into beneficial microbial resources in regard to microbial diversity and genetic polymorphisms and lays a foundation for the subsequent development and utilization of beneficial microorganisms.

## Introduction

Fermentation is the major storage method of food in many countries (Gadaga et al., 1999) and is considered a simple and inexpensive method to enhance the sensory properties and nutritional value of food, as well as to extend its shelf life (Blandino et al., 2003). A wide range of fermented food is produced around the world, such as milk (Oki et al., 2014), fruits, vegetables and seafood. Hainan Province is surrounded by a sea and has rich seafood resources, which include fish, crab, shrimp and so on, and it has a special geographical location (Peng et al., 2018) in which selection has yielded unique bacterial resources. However, fermented seafood in Hainan Province has not yet been studied. The process of traditional fermentation is generally spontaneous, triggered by microorganisms associated with the raw food materials and the external environment (Misihairabgwi and Cheikhyoussef, 2017). The unique structure of the microbiota in each fermented food described in previous studies has confirmed the microbial activity and medicinal value of fermented food products (Singh et al., 2018). Therefore, this research on the microbial diversity of fermented seafood explores the treasure trove of unique microbial resources in Hainan Province.

Probiotics were defined as “Live microorganisms which when administered in adequate amounts confer a health benefit on the host” (FAO/WHO, 2001). *Bifidobacterium* and *Lactobacillus* are the most commonly used probiotics (Yaqoob, 2014). Many fermented foods contain live microorganisms that may have good benefits to consumers’ health (Rezac et al., 2018), with *Lactobacillus* being especially varied (Gareau et al., 2010) and considered to be able to prevent gastrointestinal disorders (Tamang et al., 2016) and chronic diseases, including liver disease (Qin et al., 2014), hyperlipidaemia (Shao et al., 2017) and cardiovascular diseases (Liu et al., 2015), as well to be able to lower the risk of the type two diabetes (Rezac et al., 2018). We collected fermented seafoods, including fish sauce, shrimp sauce and crab sauce, from different areas. Fishes sauce is usually produced using tilapia, which is different from fish tea, a mixture of fish, rice and seasoning (Zhang et al., 2016). Shrimp sauce is usually produced using *Metapenaeus ensis*, and crab sauce is usually produced using hele crab (*Scylla serrate*). Fermented seafood was washed and crushed, and then 6–7% of salts were added to obtain the natural fermentation. Lactic acid was the main produced acid in the process of microbial fermentation. These sauces are semifluid, have many small solid particles and are often used as seasoning in cooking. The most commonly eaten home dish in Hainan Province is fried sweet potato leaves in shrimp sauce.

In recent years, the expansion of genomic sequencing has promoted tremendous advances in metagenomics (Dark, 2013), which have made it easier to accurately study the microbial structure and probiotic potential in samples. Bedoya et al. (2019) studied the microbial diversity in the environment of a waste water treatment plant in Colombia using next-generation sequencing techniques. The microbial diversity in naturally fermented tofu whey, a traditional Chinese tofu-coagulant, was first analyzed using high-throughput sequencing (Fei et al., 2018). In addition, interactions between bacteria and the environment play important roles in the ecological and evolutionary processes (Good et al., 2017). Therefore, different single nucleotide polymorphisms (SNPs) of bacteria are present due to the different raw materials and environment of the fermented food. The difference in SNPs could help us to understand the influence of the environment on microbial variation and provide data for directional variation of microorganisms by changing the environment. In the present study, microbial diversity and metabolic pathways in fermented vegetables in Hainan Province were studied. In this research, not only did we study the microbial diversity in fermented seafood, we also studied the difference between fermented vegetables and seafood.

## Materials and Methods

### Collection and Chemical Analysis of Samples

In this study, 23 fermented seafood samples were collected. Nine fermented fish samples were collected from the cities of Lingshui, Baoting and Qiongzhong; 5 fermented crab samples were collected from Wanning City; and 9 fermented shrimp samples were collected from the cities of Baoting, Ledong, Lingshui and Wanning in Hainan Province in China. The specific sampling areas were in the Supplementary Table S1. The sampling time was October 2017. Fermented seafoods were produced by local populations through natural fermentation and were collected from the local population after the fermentation ended. Fermentation usually ended in 10–15 days. After collection, these samples were placed in a cooler and immediately transported to the laboratory and stored at -20°C for DNA extraction. The pH value was measured for a uniform mixture of one gram of fermented seafood sample with 10 mL of a sterile NaCl solution (0.85%, w/v).

### Sample Processing and Strain Preservation

Ten grams of fermented seafood sample was mixed uniformly with 90 mL of a sterile NaCl solution (0.85%, w/v). On the one hand, the solution was used to dilute the mixture, which was then coated on MRS solid agar medium; On the other hand, the diluent of mixture was used to extract DNA directly for 16S rDNA sequencing.

A single colony was selected, and DNA was extracted after the second-generation of the culture. After being oscillated evenly, the supernatant was used for DNA extraction with a QIAGEN DNA Mini-Kit (QIAGEN, Hilden, Germany) and a bead-beating method (Tanaka et al., 2009). The quality of the extracted DNA was detected by 1% agarose gel electrophoresis, and DNA samples were stored at -20°C for further processing. The universal forward primer A27F (5′-GCAGAGTTCTCGGAGTCACGAAGAGTTTGATCCTGGCTCA-3′) and the reverse primer A1495R (5′-AGCGGATCACTTCACACAGGACTACGGCTACCTTGTTACG-3′) were used to amplify the gene. Sequencing of the single colony was performed in Shanghai Personal Biotechnology Company. The sequencing results of the single bacteria were compared with the NCBI database after being aligned with MEGA software and the identification results were obtained. After identification, the strains were stored at -80°C in the laboratory for later experiments (SRA accession: PRJNA517547).

### Sample DNA Extraction and PCR Amplification, Quantification, Pooling and Sequencing

The DNA of fermented seafood was extracted with a QIAGEN DNA Mini-Kit. The V3-V4 region of the 16S ribosomal RNA (rRNA) genes was amplified (Fouhy et al., 2016), and a set of 6-nucleotide barcodes was added to the universal forward primer 338F (5′-ACTCCTACGGGAGGCAGCA-3′) and the reverse primer 806R (5′-GGACTACHVGGGTWTCTAAT-3′) (Wang et al., 2018). The PCR products were quantified by using an Agilent DNA 1000 Kit and an Agilent 2100 Bioanalyzer (Agilent Technologies, United States). The Illumina MiSeq high-throughput sequencing platform was used to sequence the amplified products, which were pooled to a final concentration of 100 nmol/L at equimolar ratios. The 16S rDNA of fermented seafood samples were sequenced and the sequences of fermented seafood were submitted to the NCBI database (SRA accession: PRJNA507916).

### Bioinformatics and Statistical Analyses

We performed microbial community analyses in the QIIME platform (v1.7) using high-quality sequences (Caporaso et al., 2010b). PyNAST (Caporaso et al., 2010a) and UCLUST (Edgar, 2010) were used to align sequences and cluster under 100% sequence identity to obtain the unique V3-V4 sequence. Operational taxonomic units (OTUs) were classified using UCLUST after representative sequences were selected with a 97% threshold identity. The taxonomy of each OTU representative sequence was assigned using the Ribosomal Database Project (RDP) classifier with a minimum bootstrap threshold of 80% (Cole et al., 2007). A representative set of OTUs checked for chimeras and established in FastTree (Price et al., 2009) was used to construct a taxonomic tree for downstream analyses, including alpha and beta diversity calculations. To evaluate alpha diversity, the Shannon-Wiener and Simpson’s diversity indices were calculated, and the Chao1 and ACE indices were measured to estimate community richness. UniFrac metrics, which are used to measure phylogenetic distance, were calculated to evaluate the beta diversity between the sets of sequences collected from many different microbial communities, and both weighted and unweighted calculations were performed prior to principal coordinate analysis (PCoA). PICRUSt (Phylogenetic Investigation of Communities by Reconstruction of Unobserved States) was used to predict the metabolic pathways of the microbiota (Langille et al., 2013). All statistical analyses were conducted using the R program. The PCoA results were visualized with the ggplot2 package (Ito and Murphy, 2013). The relative abundance of taxa was compared by the Kruskal-Wallis test based on the rarefied OTU subset (Elliott and Hynan, 2011). The network was drawn by Cytoscape software (v3.6.0) (Killcoyne et al., 2009).

In our previous research, a diverse selection of fermented vegetables samples [including fermented Chinese cabbages (FCC), fermented bamboo shoots (FBS) and fermented watermelons (FW)] was analyzed and various bacterial strains were isolated (Peng et al., 2018). A variety of bacterial strains was also isolated from fermented seafood, and 16s rDNA was sequenced and compared with the NCBI database. Of which, the 16S rDNA sequences of 124 *Lactobacillus plantarum* and 158 *Lactobacillus fermentum* strains were chosen for a comparison of SNPs (Qi et al., 2013).

## Results

### Comparison of the Climate Conditions and pH Among Samples and Microbial Diversity of Fermented Seafood Samples

**FIGURE 1:**
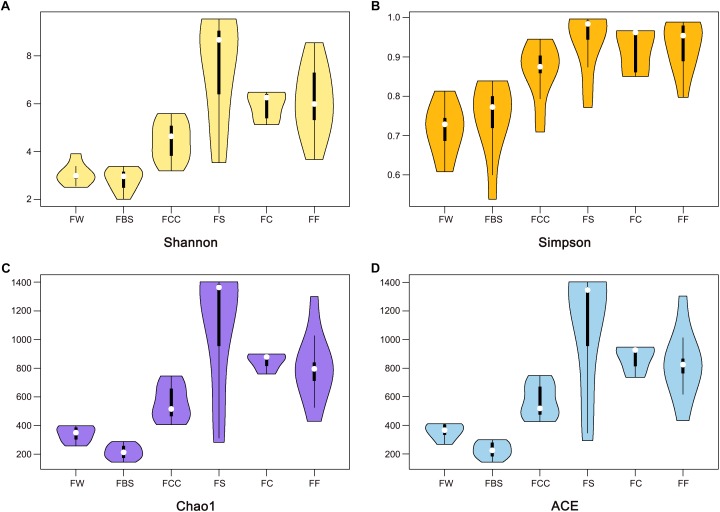
Alpha diversity of bacterial genera using the Shannon, Simpson, Chao1, and ACE **(A–D)** indices.

We measured the pH value in the samples (Table 1). Interestingly, the average pH value of fermented shrimp was dramatically higher than in the other samples (*P* < 0.001), and no significant differences were observed among FBS, FCC, FW, FC and FF. The Shannon (Figure 1A), Simpson (Figure 1B), Chao1 (Figure 1C) and ACE (Figure 1D) indices were measured to evaluate the alpha diversity of the fermented seafood samples [including fermented fish (FF), fermented crabs (FC) and fermented shrimp (FS)]. The higher alpha diversity of fermented seafood indicated that the microbial species found were more abundant than those found in fermented vegetables (*p* < 0.001). The alpha diversity of FS was highest in fermented seafood, but in the fermented seafood samples, no significant differences existed among between FS, FC and FF. This result meant that a different alpha diversity existed among the different raw materials of the fermented species.

**Table 1:** Comparison of the climate conditions and pH among samples.

	Samples	Elevation	Temperature	Humidity	pH
FV	FBS	157.38	23.20	0.79	3.56 ± 0.38
	FCC	97.32	23.83	0.80	3.89 ± 0.23
	FW	238.08	23.28	0.79	3.54 ± 0.18
FS	FC	164.26	23.44	0.79	4.08 ± 0.11
	FF	163.87	23.72	0.76	3.97 ± 0.37
	FS^∗∗∗^	206.49	23.94	0.70	7.24 ± 0.75

*^∗∗∗^P < 0.001.*

### Microbial Community Structure Diversity in the Fermented Seafood Samples

**FIGURE 2:**
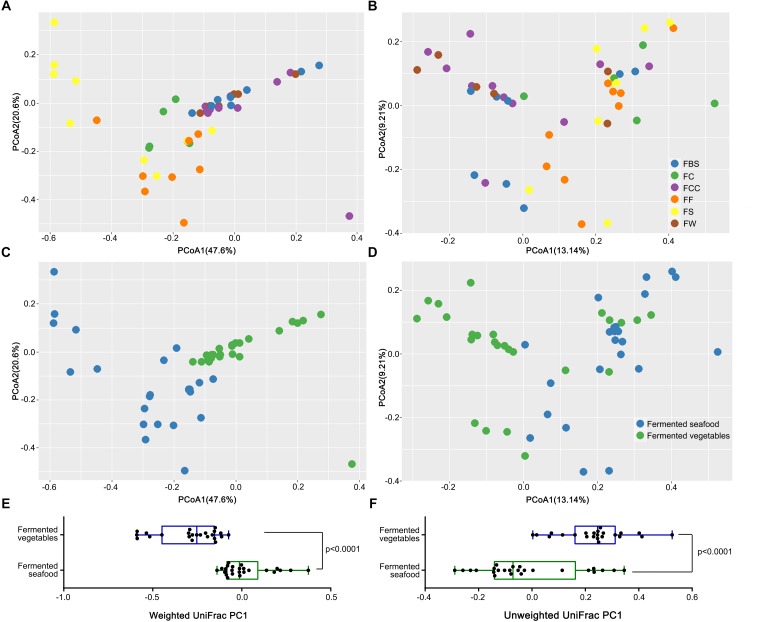
Principal coordinate analysis (PCoA) was used to evaluate the similarity between samples using the UniFrac distance. The UniFrac distance can be divided into unweighted **(B,D)** and weighted **(A,C)**. The former only considers whether an OTU exists in the sample or not, and the latter takes into account the phylogenetic relationship among community members and their abundance in the samples. The weighted UniFrac PC1 **(E)** and unweighted UniFrac PC1 **(F)** were used to make box plots respectively to compare the differences between fermented vegetables and fermented seafood. Each point represents the composition of the microbiota of one sample.

Based on principal coordinate analysis (PCoA) of the weighted UniFrac distance (Figure 2A,C) and the unweighted UniFrac distance (Figure 2B,D), we compared the intergroup differences between the fermented vegetables and fermented seafood samples to evaluate the microbial β diversity. Each point represents the microbial structure of one sample. Based on the weighted UniFrac distance, in the fermented seafood samples, the orange points of FF, the yellow points of FS, and the green points representing FC were not clearly separated (Figure 2A). However, in Figure 2C, blue points, representing fermented seafood (including FF, FS, and FC), clustered in the lower left section of the coordinate axis and green points, representing fermented vegetables (including FBS, FCC and FW), clustered in the upper right section of the coordinate axis, which showed that the structure of the microbial community was significantly different among the fermented seafood and fermented vegetable samples, and a significant separation (*P* < 0.001) in PC1 was observed based on both the weighted (Figure 2E) and unweighted (Figure 2F) Wilcoxon rank-sum tests.

### Core Microbial Genera of Fermented Seafood and Fermented Vegetables

**FIGURE 3:**
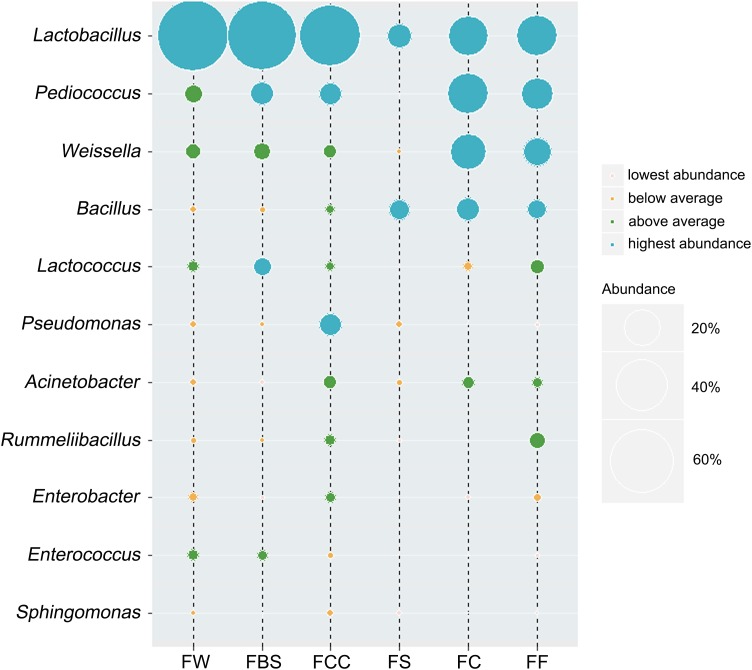
Average relative abundance of bacterial genera was more than 0.1%, and present in more than 85% of the samples.

The bacterial genera whose average relative content was more than 0.1% in all samples and were present in more than 85% of the samples were selected. We identified the dominant bacterial genera in samples (Figure 3), which included *Lactobacillus, Pediococcus, Weissella, Bacillus, Lactococcus, Pseudomonas, Acinetobacter, Rummeliibacillus, Enterobacter, Enterococcus* and *Sphingomonas*. The relative abundances of *Lactobacillus, Pediococcus, Weissella, Bacillus, Lactococcus, Pseudomonas, Acinetobacter* and *Rummeliibacillus* were more than 1%. In fermented seafood, the average abundance of *Lactobacillus* was only 19%, and in fermented vegetables, the relative average content of *Lactobacillus* was 67% (Peng et al., 2018). The *Lactobacillus*, *Pediococcus, Weissella, Caproiciproducens, Bacillus* and *Staphylococcus* contents were high in fermented seafood, and *Caproiciproducens* was a unique and dominant genus of fermented seafood, whereas only *Lactobacillus* played an absolutely leading role in fermented vegetables.

### The Comparison of Genera Among Fermented Seafood

We also compared the bacterial genera found among the FC, FF and FS. *Caproiciproducens* was the most abundant genus in FS (Table 2), and *Lactobacillus* was the most abundant genus in FC and FF. *Lactobacillus* and *Pediococcus* were dominant genera in FF and FC; interestingly, the presence of these two genera was lower in fermented shrimp, and *Lentibacillus*, *Ochrobactrum*, and *Bacterium* were more abundant in FC and FF.

**Table 2:** Comparison of the genera among FC, FF, and FS in fermented seafood.

	Average			Maximum, Minimum			
Genus	FC	FF	FS	FC	FF	FS	*P*-value
*Lactobacillus*	24.925	25.740	9.037	0.519, 0.083	0.631, 0.015	0.521, 0.008	0.0644
*Pediococcus^∗∗^*	26.060	15.410	0.094	0.441, 0.054	0.671, 0.000	0.002, 0.000	0.0051
*Weissella^∗∗^*	20.307	11.803	0.421	0.283, 0.155	0.367, 0.000	0.026, 0.000	0.0073
*Caproiciproducens^∗∗^*	0.056	4.277	13.292	0.002, 0.000	0.132, 0.000	0.385, 0.004	0.0053
*Bacillus*	8.102	5.486	6.083	0.118, 0.025	0.287, 0.001	0.466, 0.003	0.0905
*Staphylococcus*	0.070	1.387	8.388	0.001, 0.000	0.049, 0.000	0.402, 0.000	0.5955
*Gluconobacter*	0.049	5.056	0.007	0.001, 0.000	0.275, 0.000	0.000, 0.000	0.1231
*Lentibacillus^∗^*	0.356	0.061	4.830	0.008, 0.002	0.004, 0.000	0.281, 0.000	0.0208
*Clostridium_sensu_stricto_12^∗∗^*	0.007	1.140	3.866	0.000, 0.000	0.051, 0.000	0.119, 0.001	0.0038
*Ochrobactrum^∗∗^*	0.064	0.572	3.929	0.001, 0.000	0.031, 0.001	0.164, 0.001	0.0013
*Bacterium^∗∗^*	0.085	0.499	3.961	0.002, 0.000	0.031, 0.000	0.071, 0.001	0.0056
*Rummeliibacillus^∗^*	0.031	4.105	0.213	0.000, 0.000	0.330, 0.000	0.004, 0.000	0.0179
*Streptococcus^∗∗^*	6.356	0.433	0.036	0.082, 0.040	0.019, 0.000	0.002, 0.000	0.0019
*Lactococcus^∗∗^*	1.125	3.076	0.051	0.013, 0.010	0.138, 0.000	0.002, 0.000	0.0017
*Leuconostoc^∗∗^*	5.396	0.511	0.025	0.085, 0.027	0.028, 0.000	0.002, 0.000	0.0007
*Acinetobacter*	2.145	1.609	0.623	0.038, 0.004	0.062, 0.001	0.013, 0.000	0.2014
*Klebsiella^∗∗^*	0.376	1.428	0.011	0.005, 0.003	0.033, 0.000	0.000, 0.000	0.0008
*Escherichia-Shigella^∗∗^*	0.337	1.120	0.147	0.005, 0.002	0.059, 0.001	0.003, 0.000	0.0080

*^∗^P < 0.05, ^∗∗^P < 0.01.*

### Comparison of Metabolic Pathways Abundance in the Samples

**FIGURE 4:**
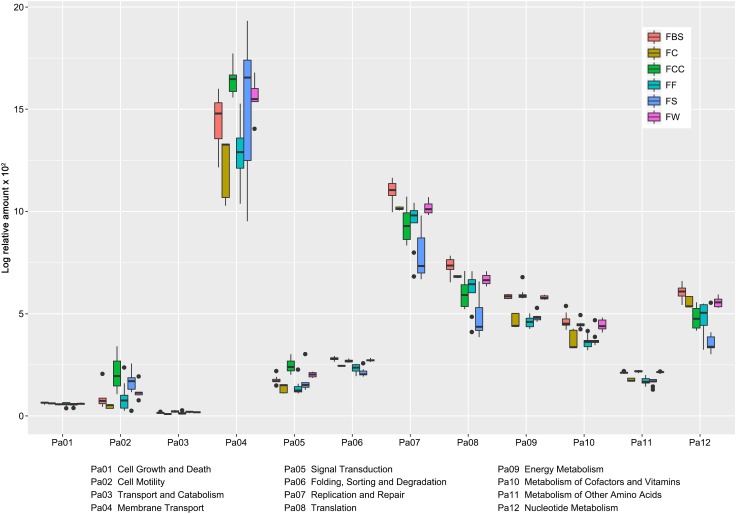
Abundance of metabolic pathways that showed differences (*P* < 0.05) among the samples was compared by using PICRUSt.

After the different microbial genera had been identified, the metabolic pathways in the samples were predicted. We selected and compared the metabolic pathways that had significant differences (*P* < 0.05) among all samples (Figure 4). The abundance of Membrane Transport, Replication and Repair, Translation, and Nucleotide Metabolism were higher than other pathways. Membrane Transport plays an important role in the endocytic pathway and transport proteins, and the metabolic pathway of Membrane Transport in FS was the highest.

### Correlation Analysis Network of the Core Microbial Genera, Metabolic Pathways, Climatic Conditions and pH Value

**FIGURE 5:**
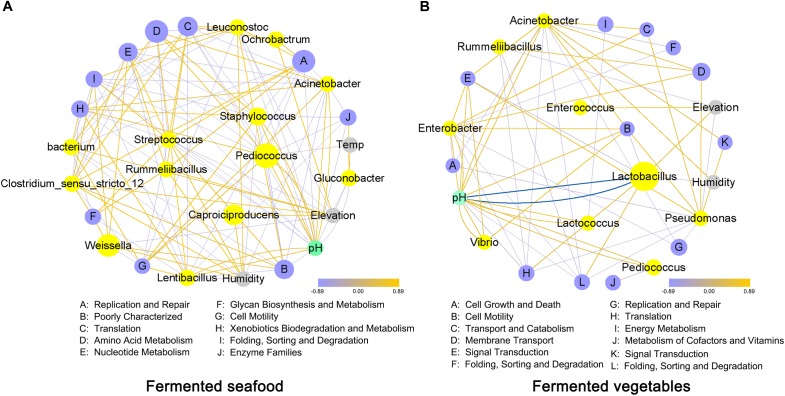
Correlation analysis of climatic conditions, bacterial genera, metabolic pathways and pH value in fermented seafood **(A)** and fermented vegetables **(B)**; correlation analysis was conducted by using the Spearman method, and the network was drawn with Cytoscape software. Data with an *r* value greater than 0.4 or *r* value less than –0.4 were selected. The color of the line connecting the different points indicates positive and negative correlations; the red line represents a positive correlation, and the blue line represents a negative correlation. The degree of thickness of the line shows the strength of the correlation, and the size of the points indicates the relative content.

We performed a correlation analysis of climatic conditions, core bacterial genera, microbial metabolic pathways and pH value in fermented seafood (Figure 5A) and fermented vegetables (Figure 5B). The relative abundances of *Pediococcus*, *Caproiciproducens* and *Weissella* were highest in fermented seafood; however, in fermented vegetables, only *Lactobacillus* had a higher abundance than other microbial genera. *Lactobacillus* had an obvious strong negative correlation with pH value, as shown in Figure 5B, which meant the high abundance of *Lactobacillus* would reduce the pH value, and this finding also demonstrated that fermented vegetables had a low pH value. The relative content of the metabolic pathways of Amino Acid Metabolism was the highest in fermented seafood, and Membrane Transport and Replication and Repair were the metabolic pathways with the highest abundance in fermented vegetables.

### 16s rDNA Identification of Bacteria in Fermented Seafood

Based on the 16S rDNA sequencing, 178 strains of bacteria were isolated and identified (Table 3). Although the microbial diversity between fermented vegetables and fermented seafood was different, *Lactobacillus fermentum* and *Lactobacillus plantarum* were still dominant. *Lactobacillus fermentum* had a higher abundance in FS, whereas *Lactobacillus plantarum* had a higher abundance in FF.

**Table 3:** 16S rDNA identification of bacteria in fermented seafood.

Identification	Samples		
	FF	FC	FS
*Enterococcus faecalis*	3/3	–	–
*Lactobacillus acidipiscis*	1/1	–	–
*Lactobacillus brevis*	–	1/1	–
*Lactobacillus buchneri*	1/1	–	–
*Lactobacillus farciminis*	–	3/3	–
*Lactobacillus fermentum*	13/54	10/54	31/54
*Lactobacillus futsaii*	–	2/2	–
*Lactobacillus namurensis*	6/6	–	–
*Lactobacillus panis*	1/1	–	–
*Lactobacillus paracasei*	3/3	–	–
*Lactobacillus pentosus*	1/5	2/5	2/5
*Lactobacillus plantarum*	37/71	18/71	16/71
*Lactobacillus pontis*	–	–	4/4
*Lactobacillus reuteri*	–	–	8/8
*Pediococcus acidilactici*	1/1	–	–
*Pediococcus pentosaceus*	–	1/6	5/6
*Staphylococcus condimenti*	–	–	2/2
*Staphylococcus epidermidis*	–	–	3/3
*Weissella cibaria*	1/1	–	–
*Weissella confusa*	2/2	–	–

### Single Nucleotide Polymorphisms (SNPs) of *Lactobacillus plantarum* and *Lactobacillus fermentum*

**FIGURE 6:**
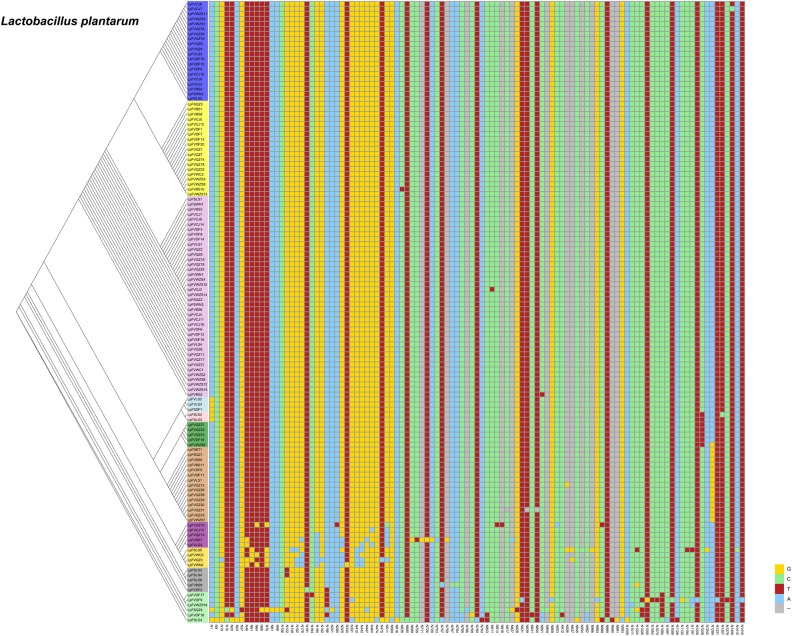
SNPs of *Lactobacillus plantarum* in the samples.

**FIGURE 7:**
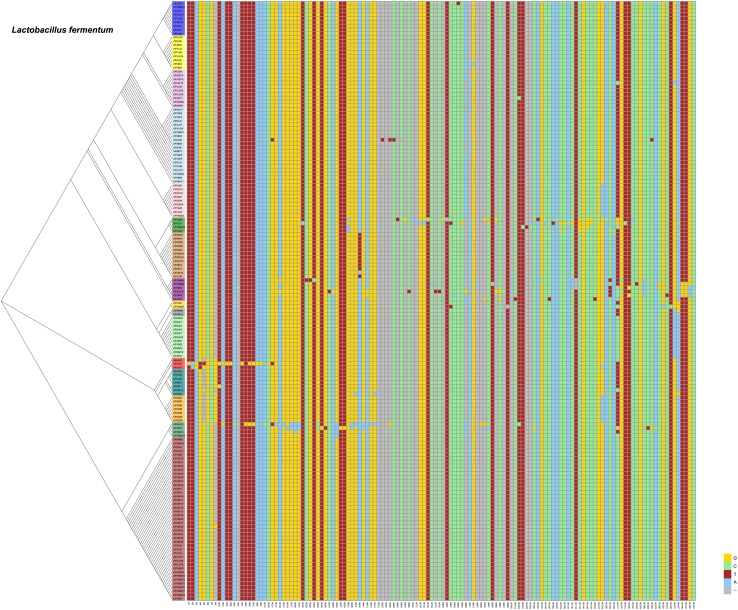
SNPs of *Lactobacillus fermentum* in the samples.

From our previous research, we found that *Lactobacillus fermentum* and *Lactobacillus plantarum* were the predominant Lactobacillus species in fermented vegetables. We wanted to determine the evolutionary distance of *Lactobacillus fermentum* and *Lactobacillus plantarum* in the different fermented foods. Thus, we compared SNPs of *Lactobacillus plantarum* and *Lactobacillus fermentum*. We found 107 SNPs from the 16S rDNA sequences of *Lactobacillus plantarum* and 134 SNPs of *Lactobacillus fermentum* in the samples (Figure 6, 7). The evolutionary tree of *Lactobacillus plantarum* and *Lactobacillus fermentum* is illustrated on the left of the figure. In Figure 6, the mutations are concentrated in the lower part, and in Figure 7, the mutations are concentrated in the middle part. Mutagenesis has been shown to occur in strains that are close to each other in evolution, and by further comparison, we found that most of the mutations occurred in fermented vegetables.

## Discussion

Fermented products play an important role in human life and diet, such as fermented suan-cai from Northeast China (Yu et al., 2015), yogurt (Parvez et al., 2006), saeu-jeot (Jung et al., 2016) and so on. Traditionally, fermented food result from natural fermentation, which is affected by climate, humidity, and geographical location. In recent years, research on the microbial composition and community structure in fermented food has been greatly improved by the development of metagenomics (Kergourlay et al., 2015) based on next-generation high-throughput sequencing technology. Li et al. (2017) investigated the prokaryotic community succession and metabolite changes of doubanjiang-meju, a major ingredient of Chinese fermented food, using the high-throughput sequencing method. Lee, Se-Hui et al. identified the diversity and community of fermenting bacteria isolated from eight major Korean fermented foods using a metagenomic approach (Lee et al., 2015).

Our study examined the alpha diversity and microbial community structure of fermented seafood based on a high-throughput sequencing technology and compared fermented vegetables and fermented seafood to determine differences. The alpha diversity of fermented seafood was higher than that of fermented vegetables and attained the highest level in fermented shrimp, which was also demonstrated through the presence of different genera in fermented vegetables compared to fermented seafood. The type of raw material, production location and climate conditions had an influence on the diversity (Nguyen et al., 2013), and we found that the species in the raw materials had a greater influence than other factors described in a previous work (Peng et al., 2018).

The pH value in fermented shrimp was higher than in the other samples. On one hand, during the progression of shrimp fermentation, protein catabolism produces ammonia, and the contents of amino nitrogen are increased in the fermentation period (Xiao-Xi et al., 2015), which may lead to the rise in the pH value. Membrane Transport and Amino Acid Metabolism were the abundant metabolic pathways in fermented shrimp, indicating that protein and amino acid metabolism were abundant. On the other hand, shrimp contained a high amount of proteins, lipids, and astaxanthin pigment carotenoids, peptides and free amino acids (Ruttanapornvareesakul et al., 2005). The astaxanthin content was more abundant in shrimp than in fish, and astaxanthin had a good antioxidant ability (Sang and Min, 1990), inhibiting lipid oxidation to produce CO_2_, which might inhibit the decrease in the pH value. Furthermore, *Lactobacillus* was the overwhelmingly dominant genus in the other fermented foods and can ferment sugars in vegetables to produce acid, which might decrease the pH value (Peng et al., 2018).

We analyzed the SNPs of *Lactobacillus plantarum* and *Lactobacillus fermentum* and found that most of the mutations occurred in fermented vegetables. Mutations were associated with environmental factors (Parajuli et al., 2018) and pharmacological and toxicological effects (Uno et al., 2018). In the edible portion, most varieties of vegetables are mostly composed of water and have approximate mean sugar values (glucose + fructose + sucrose) between 0.5 and 4.5%, as well as vitamins, minerals, and a small amount of protein (Somogyi and Trautner, 1974). In seafood, protein and fat were more abundant than in vegetables, estimated at 20% each (Aberoumand, 2012; Domingo, 2016), and the glutamic acid abundance was greatest in seafood (Jang et al., 2013). Thus, seafood could provide more needed and readily available nutrients than vegetables for microorganisms, and the competition for nutrients from bacteria in fermented vegetables would have a higher selection pressure than in fermented seafood. Good et al. found that adaptation to the environment can be a complex and dynamic process, driven by the accumulation of mutations, with variants that are beneficial, competing for dominance in each population (Good et al., 2017). Fermented vegetables might provide a stronger environmental and competitive pressure for bacteria, which might lead to more mutations.

In this study, the microbial diversity of fermented seafood in the Hainan area was systematically studied. This research showed that fermented seafood had high alpha diversity and that the microbial structure was different. Based on our previous research, we also compared the differences between fermented seafood and fermented vegetables; those differences were mainly reflected in the alpha and beta diversities and the dominant microbial genera. Fermented shrimp had the highest alpha diversity, and the dominant bacteria were also different. Furthermore, we analyzed the 16S gene polymorphisms of the same dominant species (*Lactobacillus plantarum* and *Lactobacillus fermentum*) in two fermented environments, which showed that most of the mutations occurred in fermented vegetables. The raw materials had a greater impact on the microbiota in the fermented products. This research provides new insight into the beneficial microbial resources in regard to microbial diversity and genetic polymorphisms, providing basic research data for the subsequent development and utilization of beneficial microorganisms.

## Data Availability

The sequence data reported in this paper have been submitted to the NCBI database (Accession No. PRJNA507916).

## Author Contributions

JZ and WL developed the experimental design. SJ and CM conducted the research. DH provided essential reagents. CM and QP analyzed the data. SJ and QP wrote the manuscript.

## Conflict of Interest Statement

The authors declare that the research was conducted in the absence of any commercial or financial relationships that could be construed as a potential conflict of interest.

## Supplementary Material

TABLE S1 The sampling sites of fermented seafood.
